# Supplementary figures and images for: Analyzing the interpretative ability of landscape pattern to explain thermal environmental effects in the Beijing-Tianjin-Hebei urban agglomeration
Source: PeerJ. 2019 Oct 7;7:e7874. doi: 10.7717/peerj.7874 (PMC6786252; doi:10.7717/peerj.7874)

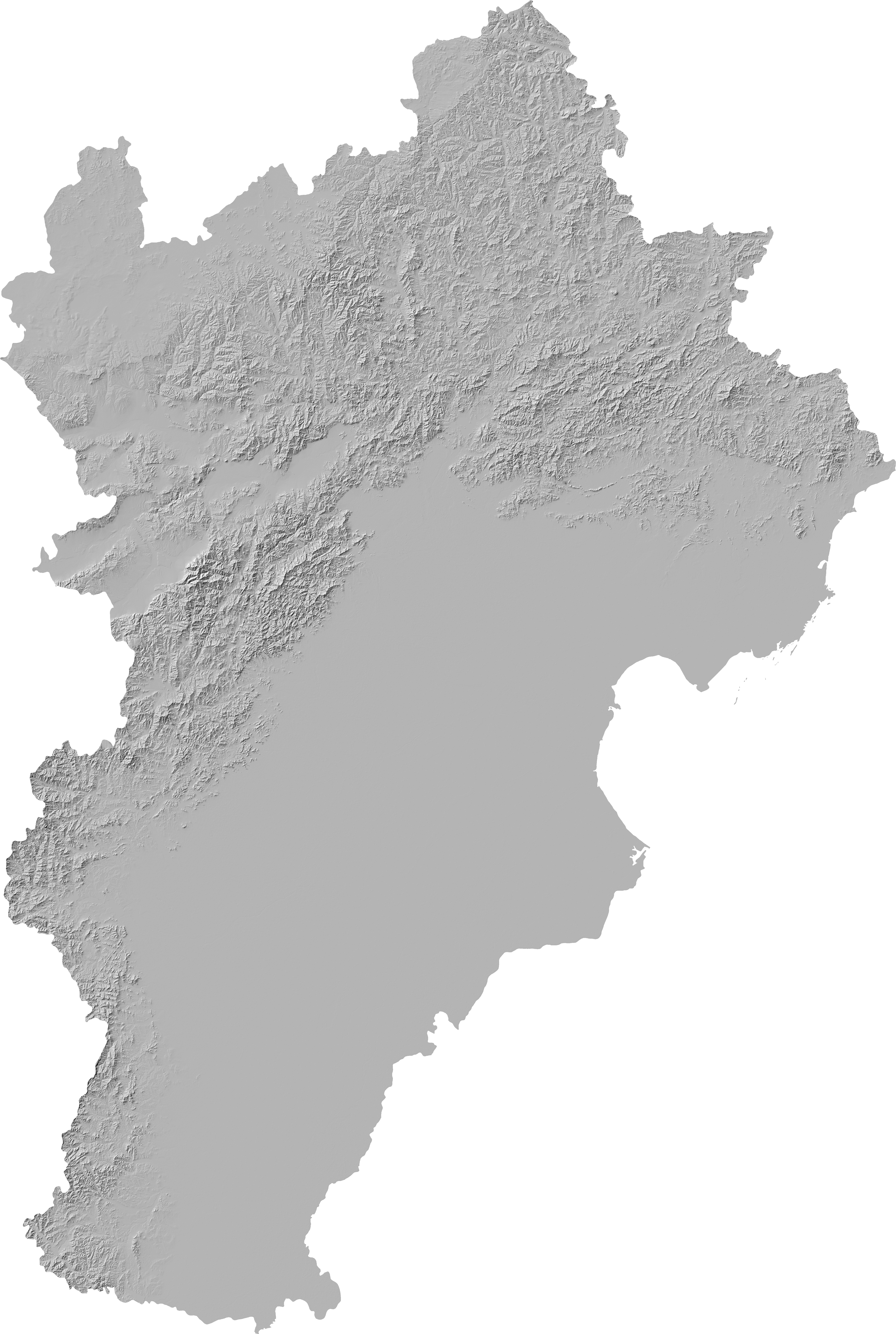

Supplement: Supplemental Information 7 — It was generated from DEM data. [file peerj-07-7874-s007.zip › hillshade/╔╜╥⌡╙░╡╪╨╬1.tif]

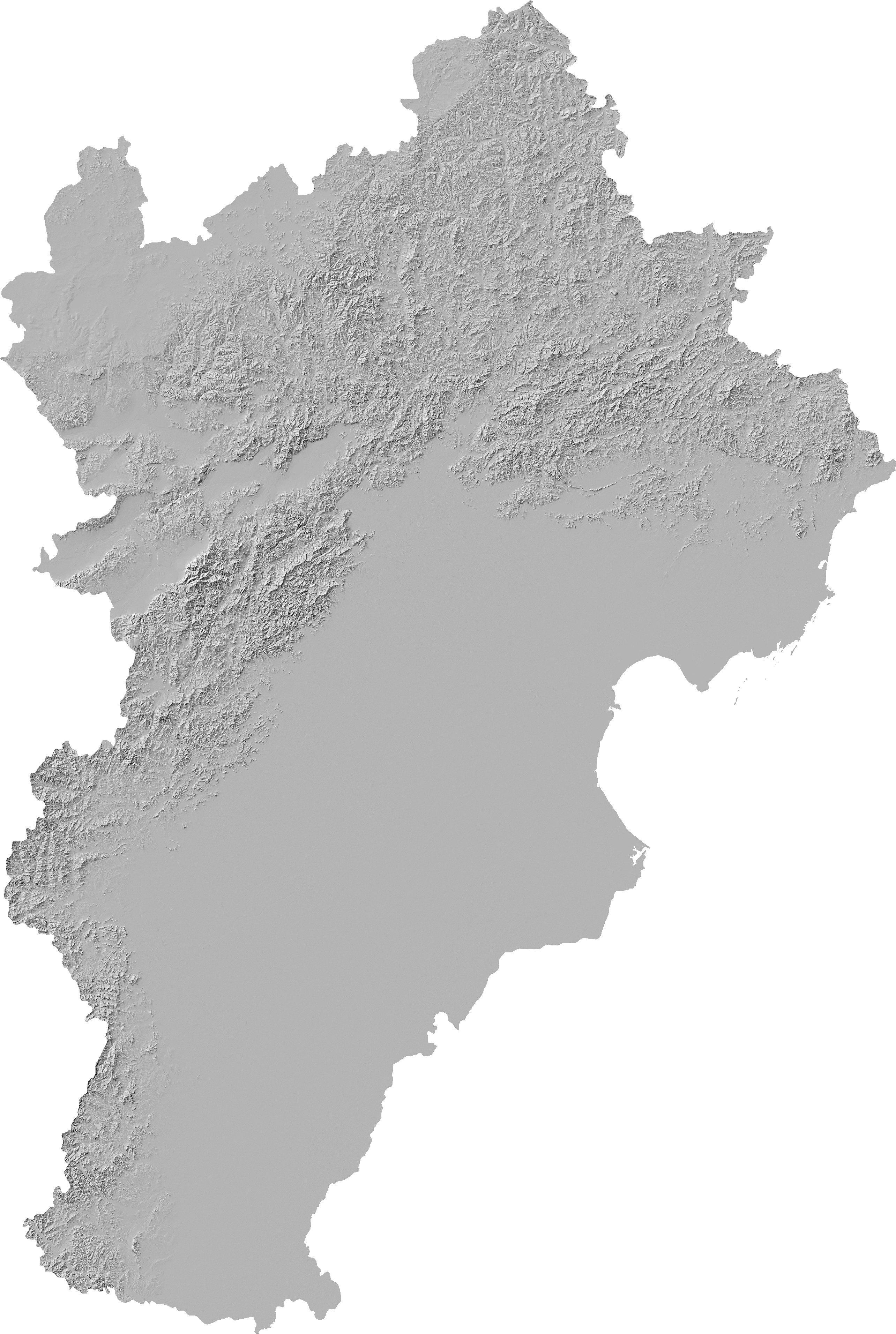

Supplement: Supplemental Information 7 — It was generated from DEM data. [file peerj-07-7874-s007.zip › hillshade/╔╜╥⌡╙░╡╪╨╬1.tif.ovr]
